# What type, or combination of exercise can improve preferred gait speed in older adults? A meta-analysis

**DOI:** 10.1186/s12877-015-0061-9

**Published:** 2015-07-01

**Authors:** Renske Van Abbema, Mathieu De Greef, Celine Crajé, Wim Krijnen, Hans Hobbelen, Cees Van Der Schans

**Affiliations:** 1 Research group Healthy Ageing, Allied Health Care and Nursing – Hanze University Groningen, University of Applied Sciences, PO Box 3109, 9701 DC Groningen, The Netherlands; 2 Institute of Human Movement Sciences, University of Groningen, Groningen, The Netherlands; 3 Department of Rehabilitation Medicine, Center for Rehabilitation, University Medical Center Groningen, Groningen, The Netherlands

**Keywords:** Systematic review, Elderly, Gait speed, Exercise

## Abstract

**Background:**

Improved preferred gait speed in older adults is associated with increased survival rates. There are inconsistent findings in clinical trials regarding effects of exercise on preferred gait speed, and heterogeneity in interventions in the current reviews and meta-analyses.

*Objective:* to determine the meta-effects of different types or combinations of exercise interventions from randomized controlled trials on improvement in preferred gait speed.

**Methods:**

*Data sources:* A literature search was performed; the following databases were searched for studies from 1990 up to 9 December 2013: PubMed, EMBASE, EBSCO (AMED, CINAHL, ERIC, Medline, PsycInfo, and SocINDEX), and the Cochrane Library.

*Study eligibility criteria:* Randomized controlled trials of exercise interventions for older adults ≥ 65 years, that provided quantitative data (mean/SD) on preferred gait speed at baseline and post-intervention, as a primary or secondary outcome measure in the published article were included. Studies were excluded when the PEDro score was ≤4, or if participants were selected for a specific neurological or neurodegenerative disease, Chronic Obstructive Pulmonary Disease, cardiovascular disease, recent lower limb fractures, lower limb joint replacements, or severe cognitive impairments. The meta-effect is presented in Forest plots with 95 % confidence

*Study appraisal and synthesis methods:* intervals and random weights assigned to each trial. Homogeneity and risk of publication bias were assessed.

**Results:**

Twenty-five studies were analysed in this meta-analysis. Data from six types or combinations of exercise interventions were pooled into sub-analyses. First, there is a significant positive meta-effect of resistance training progressed to 70-80 % of 1RM on preferred gait speed of 0.13 [CI 95 % 0.09-0.16] m/s. The difference between intervention- and control groups shows a substantial meaningful change (>0.1 m/s). Secondly, a significant positive meta-effect of interventions with a rhythmic component on preferred gait speed of 0.07 [CI 95 % 0.03-0.10] m/s was found. Thirdly, there is a small significant positive meta-effect of progressive resistance training, combined with balance-, and endurance training of 0.05 [CI 95 % 0.00-0.09] m/s. The other sub-analyses show non-significant small positive meta-affects.

**Conclusions:**

Progressive resistance training with high intensities, is the most effective exercise modality for improving preferred gait speed. Sufficient muscle strength seems an important condition for improving preferred gait speed. The addition of balance-, and/or endurance training does not contribute to the significant positive effects of progressive resistance training. A promising component is exercise with a rhythmic component. Keeping time to music or rhythm possibly trains higher cognitive functions that are important for gait.

*Limitations:* The focus of the present meta-analysis was at avoiding as much heterogeneity in exercise interventions. However heterogeneity in the research populations could not be completely avoided, there are probably differences in health status within different studies.

**Electronic supplementary material:**

The online version of this article (doi:10.1186/s12877-015-0061-9) contains supplementary material, which is available to authorized users.

## Background

Preferred gait speed has proven to be a strong predictor for adverse health related events in older adults [1]. Reduced preferred gait speed is associated with a higher risk for falls, disability, hospitalization, and increased mortality in both frail and well-functioning healthy older persons [2–4]. Preferred gait speed of less than 1.0 m/s signifies persons being at higher risk of poor health-related outcomes [3]. The causes of decreasing gait speed are not clear, however, age related disease, back or leg pain, poor vision, low levels of physical activity, low aerobic capacity, cognitive impairment, depression, and precedent falls were negatively associated with gait speed [5–7].

In positive contrast: improved gait speed is associated with increased survival rates in older adults [4]. In a pooled analysis of 9 cohort studies, survival increased significantly in increments of 0.1 m/s [8]. Additionally, in a prospective cohort study, preferred gait speed was the only physical performance measure that predicted a substantial reduction in mortality [9]. This association was consistent across different subgroups based on age, ethnicity, initial gait speed, and hospitalization.

Therefore, interventions that can improve preferred gait speed are important, and research is needed to identify successful interventions. Gait speed is sensitive to change over time. Recommended criteria for clinically meaningful change when measuring the preferred walking speed of community dwelling older adults measured over 4 or 10 m is 0.05 m/s for small meaningful change and 0.1 m/s for substantial meaningful change [10, 11]. Exercise plays an important role in improving gait speed in older adults, and there are many trials investigating the effect of exercise on gait speed; however, results are not consistent and the content of exercise interventions is very heterogeneous with regard to modality, dose, and intensity. A meta-analysis on the effect of exercise on gait speed in community dwelling elderly people included studies from 1995 to 2003 [12]. This meta-analysis included trials with different levels of evidence and quality. The authors concluded that high-intensity exercise can improve preferred gait-speed, with strength training or combination training (addition of aerobic exercise) as promising modalities. However, the overall change of 0.01-0.02 m/s was too small to be clinically meaningful.

In addition, another two reviews performed a small meta-analysis on the effect of exercise on gait speed in frail older populations. Chou et al. [13] showed a significant increase in gait speed of 0.07 m/s compared with a control group (95 % CI, 0.02 - 0.11; P = .005), and the results of Giné-Garriga et al. [14] show a preferred gait speed that was 0.06 m/s higher than in the control group (95 % CI, 0.04 - 0.08; P < .001) . However, the included studies of Chou et al. [13] use different paces (fast or preferred gait speed) in the gait speed tests, what could have influenced the mean gait speed performance. In both studies, the exercise interventions that are compared are very heterogeneous; varying from stretching to interdisciplinary interventions with a physiotherapy component. Finally, there is limited information regarding whether improvement in gait speed can be maintained after exercise interventions had ended, because of limited long term data on maintenance of gait speed from randomized controlled trials and reviews [12, 13]. More knowledge is needed on the course of gait speed over time, and what is needed to maintain the benefits from training.

In summary, improving gait speed in older adults is important, however there are inconsistent findings in clinical trials regarding effects of exercise on gait speed, and there is much heterogeneity in gait speed tests and interventions in the current reviews and meta-analyses. Strength training or a combination of strength- with aerobic training seems promising, and there are many more modalities investigated like balance-, functional-, and flexibility training. However, we only have limited time to effectively exercise with this target population. Therefore, it is important to learn if we should focus on strength training alone, or also invest time in another type of exercise modality that contributes to the results. We emphasize the need for a large updated meta-analysis. The main objective of this study was to determine the meta-effects of different types or combinations of exercise interventions from randomized controlled trials on improvement in preferred gait speed. We hypothesize that progressive resistance training has significant effect on preferred gait speed. Furthermore, a combination with balance, or endurance training may enhance this effect.

## Methods

This study is reported according to the Preferred Reporting Items for Systematic reviews and Meta-Analyses (PRISMA) guidelines [15]. The PRISMA checklist is provided in Additional file 1.

### Search strategy

A systematic review was performed to identify randomized controlled trials investigating the effect of exercise interventions on preferred gait speed in older adults. The following databases were searched: PubMed, EMBASE, EBSCO (AMED, CINAHL, ERIC, Medline, PsycInfo, and SocINDEX), and the Cochrane Library. A search strategy was designed using keywords, mesh terms, and free text words such as aged, frail elderly, randomized controlled trial, exercise, and gait speed. The Pubmed search-strategy is shown in Additional file 2. The search results were limited by the study design (randomized controlled trials). The years considered were from 1990 up to 9 December 2013. Additionally, reference lists of previous reviews and trials were searched.

### Eligibility criteria

Inclusion criteria were randomized controlled trials of exercise interventions including adults aged 65 years and older. Exercise is defined as a subset of physical activity that is planned, structured, repetitive, and purposeful in the sense that improvement or maintenance of physical fitness is the objective [16].

We included studies that compared an exercise intervention with no intervention (usual activity) or a control type of intervention consisting of general health education classes, general stretching, or social visits. We only included control interventions that performed general or upper body stretching exercise not aiming to specifically increase range of motion in hips and ankles in order to improve step length, and thereby gait speed [17, 18].

Additionally, the published article had to provide quantitative data (mean/SD) on preferred gait speed at baseline and post-intervention, as a primary or secondary outcome measure in the published article. Another criteria was that gait speed was not significantly different between the intervention, and control group at baseline. The quality of the studies was assessed with the PEDro scale; studies with a score of four or less were excluded from the meta-analysis.

We excluded studies where participants received other interventions in addition to exercise that could have influenced physical function (for example: protein supplementation, nutrition intervention, or multidisciplinary treatment). Studies were excluded when solely using a treadmill gait speed test, a gait speed test with a load, a turn or with a course longer than 30 m, because these tests measure other skills besides gait speed. Furthermore, studies were excluded in which participants were selected for a specific neurological or neurodegenerative disease, Chronic Obstructive Pulmonary Disease, cardiovascular disease, recent lower limb fractures, lower limb joint replacements, or severe cognitive impairments.

### Study selection

Two reviewers (RA and CC), screened titles and abstracts of the retrieved studies for potential relevant content by using the predetermined inclusion/exclusion criteria. Disagreements regarding inclusion were discussed until consensus was reached. When no consensus was reached, a third person was involved. Full text articles were assessed for eligibility by the first author.

### Methodological quality assessment of included trials

The methodological quality of the trials was independently assessed by two reviewers (RA and CC) using the PEDro-scale [19]. The risk of bias was assessed according to ten criteria: random allocation, concealed allocation, similar baseline characteristics of groups, subject blinding, therapist blinding, assessor blinding, measures of at least one key outcome obtained from at least 85 % of the subjects, subjects receiving treatment as allocated for or ‘intention to treat’ analysis was performed, reporting of between group statistics of at least one key outcome, and both point measures, and measures of variability are reported for at least one key outcome. Trials were rated on the basis of what information they reported. When a trial did not report if a particular criterion was met, it was scored as if the criterion was not met. Studies with a PEDro score of 4 or less were excluded from the meta-analysis, therefore studies with moderate to high quality were included [19].

### Data extraction

Data extraction was performed independently by two investigators (RA and MG). Gait speed in meters per second (m/s) was the main outcome variable of interest. Post-intervention gait speed data were gathered for intervention and control groups, allowing comparison in the assumption that groups are similar at baseline regarding important prognostic indicators in randomized controlled trials. To provide uniform data for the meta-analyses, recalculations were made from m/min to m/s, from cm/s to m/s, and when time was reported for the total walking track, those data were also recalculated to m/s.

Starting protocol, pace or length of walking tracks may have an impact on the interpretation of intergroup comparisons of gait performance. However, in a review of Graham et al. [20], only pace (fast or preferred gait speed) seems to have an influence on mean gait speed performance. Neither starting protocol nor distance seemed to have significant influence on mean gait speed [20]. In this study, only preferred gait speed (PGS) was retrieved from the studies. Furthermore, population and intervention characteristics were retrieved from the studies.

### Statistical analysis

The analyses were performed using the R statistical programming system (R Development Core Team 2013; http://www.r-project.org/). A meta-analysis was performed if data from at least three comparable interventions were available. The meta-analyses of the means and standard deviations from the trials were based upon a random-effects model in order to account for heterogeneity caused by variability among participants, place and date of the experiment, type of exercise intervention, and outcome definitions. The function ‘metacont’ of the meta library from the statistical programming language R was used to perform the meta analysis. The between study variance (tau squared), was estimated by restricted maximum likelihood, and its significance are taken to test homogeneity of variances. In case homogeneity is not rejected a common effect seems likely to be present, and differences between individual studies are a consequence of sampling variation. In case of heterogeneity, individual difference between studies may be due to methodological or clinical differences [21]. The meta-effect estimated by the inverse variance method, is presented in a Forest plot with 95 % confidence intervals, and random weights assigned to each trial. Testing the significance of the meta-effect is equivalent with the observation whether zero is contained in the confidence interval. A clinically meaningful change in preferred gait speed is considered to be small when improvement of 0.05 m/s is present, and a considerable improvement is a change of 0.10 m/s [10]. An additional analysis is performed to assess the influence of each study [22]. Funnel plots are used to assess the risk of publication bias for each meta-analysis [23, 24].

To see if other variables could be responsible for the difference in gait speed after the interventions, a *t*-test was performed after the meta-analyses. Mean exercise doses, mean age, and mean baseline gait speed were compared, between the interventions with a post-intervention gait speed under the meta-effect, and the ones above the meta-effect.

## Results

### Literature search

**Fig. 1:**
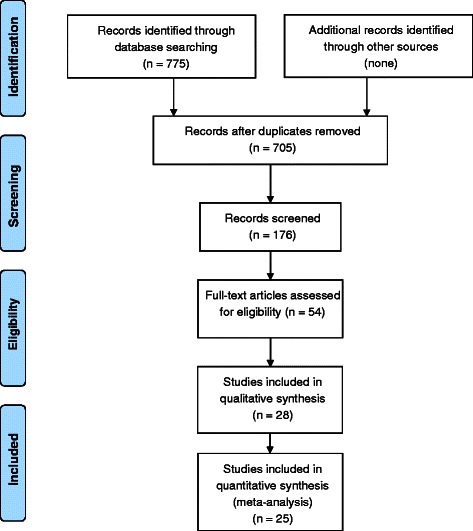
Flow chart of the study identification process

The literature search strategy yielded 705 potentially eligible articles. The identification process is presented in Fig. 1. After screening the title and the abstract, 54 articles were selected for further review of the full-text. Twenty-six of those were excluded because of the following reasons: two did not report randomized controlled trials; 13 did not have preferred gait speed data available; five involved a control group performing an exercise intervention; two involved an exercise intervention plus an additional intervention possibly influencing physical functioning; three involved a gait speed test with a load or a turn; and one reported contradicting results in the text, and displayed table. Twenty-eight articles were included in the qualitative analysis. Three articles scored four or less out of ten on the PEDro scale and were excluded from quantitative analyses (Additional file 3) [25–27].

### Study characteristics

Of the 25 analysed studies, 18 were published within the last ten years. In total, 2389 individuals participated in the interventions, with a mean age of 75,8 years. Population characteristics are presented in Table 1. Two studies involved residents of long-term care institutions [28, 29], all other studies involved community dwelling, older adults. The analysed studies were held in Europe, Australia, USA, Canada, Japan, and Brazil. Most studies compared one intervention group with a control group, however, two studies involved two intervention groups [30, 31], and one study involved three intervention groups [32]. In total, 29 interventions from 25 studies were included in the analyses. The intervention characteristics are presented in Table 2. Control groups continued their normal activities, or were provided with an attention control intervention like health-, wellness-, or driver education classes, general stretching programs, relaxation classes, or upper body strength training. Some of the studies involved single component exercise such as (progressive) resistance training, Tai Chi, balance training, salsa-dancing training, or agility training. The remaining studies involved multicomponent exercise. Nearly all interventions were supervised, only one study was home-based [33]. The total period of intervention ranged from 9 up to 48 weeks.

**Table 1:** Population characteristics of the studies included in qualitative analysis

	Study	Study population /Inclusion criteria	N (♀women/♂men)	Age mean (SD)	Gait speed Control Group		Gait speed Intervention Group	
					(m/s) Mean (SD) Baseline Posttest		(m/s) Mean (SD) Baseline Posttest	
*1*	*Arai (2007)* [37]	Community dwelling older adults > 65 years who were ambulatory with or without assisting device (Japan)	171 (?♀/?♂)	74.1	1.24 (0.21)	1.26 (0.20)	1.28 (0.24)	1.30 (0.22)
*2*	*Baker (2007)* [40]	Community dwelling older adults ≥ 60 years, residents in the retirement villages (Australia)	38 (24♀/ 14♂)	76.6 (6.1)	1.19 (0.23)	1.16 (0.25)	1.23 (0.28)	1.12 (0.23)
*3*	*Barnett (2003)* [39]	Community dwelling with one or more risk factors for falls (Australia)	163 (109♀/ 54♂)	74.9 (5.5)	0.97 (0.35)	0.98 (0.38)	0.95 (0.30)	0.98 (0.30)
*4*	*Beling (2009)* [27] *(Not included in meta-analysis PEDro < 5)!*	Community dwelling older adults ≥ 65 years, MMSE* score ≥ 24/30, complete a Timed Up and Go test ≥ 13.5 s, and/or have 2 or more falls in the past year or 1 fall with injury (Canada)	23 (11♀/ 12♂)	80.0 (5.8)	0.90 (0.22)	0.91 (0.21)	0.88 (0.20)	0.95 (0.22)
*5*	*Cress (1999)* [34]	Community dwelling older adults ≥70 years (USA)	56 (♀/♂)	75.8 (4.4)	1.37 (0.30)	1.36 (0.16)	1.46 (0.20)	1.52 (0.13)
*6*	*Doi (2013)* [41]	Community dwelling older adults ≥ 65 years with Mild Cognitive Impairment (MMSE score between 24 and 30 and memory impairment (Japan)	50 (23♀ / 27♂)	76.1 (7.2)	1.10 (0.20)	1.26 (0.21)	1.10 (0.32)	1.38 (0.32)
*7*	*Fiatarone (1994)* [29]	Nursing home residents (long term care) >70 years (USA)	100 (63♀/37♂)	87.1 (0.6)	0.47 (0.20)	0.45 (0.10)	0.51 (0.20)	0.55 (0.10)
*8*	*Freiberger (2007)* [30]	Community dwelling older adults ≥70 years (Germany)	217 (97♀/ 45♂)	75.9 (4.0)	1.30 (0.30)	1.30 (0.30)	1)1.30 (0.30)	1)1.30 (0.20)
							2)1.40 (0.30)	2)1.40 (0.20)
*9*	*Freiberger (2012)* [32]	Community dwelling older adults ≥70 years, having fallen in the past 6 months or with fear of falling (Germany)	280 (122♀/ 158♂)	76.1 (4.1)	0.95 (0.27)	0.97 (0.18)	1)0.95 (0.22)	1)0.92 (0.22)
							2)0.98 (0.18)	2)1.00 (0.17)
							3)0.98 (0.20)	3)0.95 (0.20)
*10*	*Gine-Garriga (2010)* [38]	Physically frail home-dwelling persons >10 s. on rapid gait test / not able to make 5 chair stands with hands folded or self-reported exhaustion (Spain)	51 (31♀/ 20♀)	84.0 (2.0)	0.82 (0.04)	0.80 (0.04)	0.82 (0.04)	0.94 (0.04)
*11*	*Granacher (2012)* [45]	Community dwelling older adults between 63–82 years (Germany)	28 (17♀/ 11♀)	70.8 (5.0)	1.42 (0.14)	1.42 (0.14)	1.34 (0.20)	1.49 (0.26)
*12*	*Granacher (2013)* [49]	Community dwelling older adults between 63–80 years without prior experience with core stability training (Germany)	32 (17♀/ 15♀)	70.5 (4.3)	1.42 (0.15)	1.42 (0.19)	1.41 (0.14)	1.53 (0.14)
*13*	*Halvarsson (2011)* [47]	Healthy community dwelling older adults > 65 with fear of falling and/or an experience of a fall during the previous 12 months, ability to walk unaided indoors and a MMSE score ≥24. (Sweden)	58 (42♀/17♂)	76	1.09 (0.22)	1.10 (0.23)	1.11 (0.24)	1.20 (0.17)
*14*	*Hartmann (2009)* [43]	Community dwelling older adults > 65 (Switzerland)	42 (28♀/ 14♂)	76.0 (5.8)	1.33 (0.19)	1.27 (0.14)	1.34 (0.19)	1.41 (0.19)
*15*	*Kerrigan (2003)* [17]	Healthy community dwelling older adults ≥ 65 (USA)	96 (66♀/ 30♂)	?	1.19 (0.17)	1.23 (0.18)	1.19 (0.18)	1.23 (0.18)
*16*	*Kim (2011)* [42]	Community dwelling elderly women with multiple symptoms of geriatric syndrome ≥ 70 (Japan)	61♀	78.6 (4.2)	1.20 (0.20)	1.10 (0.30)	1.10 (0.30)	1.10 (0.30)
*17*	*Lazowski (1999)* [28]	Residents of long-term care institutions with the ability to stand with minimal assistance, follow simple instructions/ demonstrations (Canada)	68 (59♀/ 9♂)	80.0 (0.9)	0.57 (0.27)	0.61 (0.31)	0.69 (0.28)	0.73 (0.33)
*18*	*Lord (1996)* [26] *(Not included in meta-analysis PEDro < 5)!*	Community-dwelling women 60-years and older	160♀	71.1 (5.2)	1.15 (0.19)	1.12 (0.18)	1.12 (0.19)	1.18 (0.18)
*19*	*Liu-Ambrose (2004)* [31]	Elderly women with osteoporosis or osteopenia (Canada)	98♀	79.0 (3.0)	0.91 (0.20)	1.00 (0.19)	1)1.02 (0.25)	1)1.11 (0.22)
							2)1.02 (0.19)	2)1.09 (0.19)
*20*	*Lustosa (2011)* [36]	Thirty-two women, over 65 years old, community-dwelling, without restriction regarding race and/or social class, classified as pre-frail according to the criteria established by Fried et al. were selected (Brazil)	32♀	72.0 (3.8)	1.22 (0.22)	1.23 (0.16)	1.24 (0.14)	1.38 (0.16)
*21*	*Persch (2009)* [35]	Elderly women (aged 60 years and over) attending local community meetings in the vicinity of the University (Brazil)	27♀	61.4 (5.5)	1.09 (0.11)	1.08 (0.14)	1.10 (0.03)	1.23 (0.07)
*22*	*Tiedemann (2013)* [50]	Community-dwelling older adults (Australia)	54 (43♀/ 11♂)	67.5 (6.6)	1.60 (0.24)	1.43 (0.21)	1.54 (0.23)	1.67 (0.17)
*23*	*Topp (1996)* [25] *(Not included in meta-analysis PEDro < 5)!*	Community-dwelling older adults (USA)	42 (33♀/ 19♂)	71.5 (1.2)	1.22 (0.14)	1.24 (0.11)	1.24 (0.14)	1.28 (0.06)
*24*	*Trombetti (2010)* [44]	Community-dwelling individuals older than 65 years, who are at increased risk of falling (Switzerland)	134 (129♀/ 5♂)	75.5 (7.0)	1.02 (0.19)	1.04 (0.13)	1.04 (0.19)	1.10 (0.13)
*25*	*Watt (2011)* [18]	Healthy older adults with aged 65 years and older (USA)	82	72.6 (6.0)	1.22 (0.21)	1.22 (0.19)	1.31 (0.25)	1.33 (0.24)
*26*	*Watt (2011)* [18] *Frail Elderly*	Frail elderly with (1) a low Instrumental Activities of Daily Living score (<3/5); (2) a major orthopedic diagnosis in the lower back, pelvis, or lower extremities since the age of 50 years; or (3) a performance on a Mini Mental Status Examination of less than 24 out of 30 (USA)	100	77.0 (8.0)	1.10 (0.20)	1.10 (0.20)	1.15 (0.2)	1.20 (0.20)
			74 analyzed (40♀/ 34♂)					
*27*	*Wolf (2006)* [48]	Transitionally frail older adults from independent living facilities with 4 frail and no more than 1 vigorous attributes according to the criteria of Speechly & Tinetti (USA)	212 (192♀/ 20♂)	80.9	0.94 (0.49)	0.99 (0.45)	1.01 (0.48)	1.08 (0.44)
*28*	*Yang (2011)*	Older people (>65 years) who reported concerns about their balance but remained community ambulant (Australia)	165 (73♀/ 92♂)	80.6 (6.2)	1.09 (0.22)	1.04 (0.23)	1.02 (0.26)	1.02 (0.22)

**Table 2:** Intervention characteristics of the included studies per sub analysis

*PROGRESSIVE RESISTANCE TRAINING*									
Study Ascending effect	Intervention	Duration and frequency	Total doses (minutes)	Intensity	Baseline gait speed (mean (SD))	Gait speed test	Exercise compliance	Mean age	Pedro score
*Fiatarone (1994)* [29]	Progressive resistance exercise training of hip and knee extensors	10 weeks, 3x p/w; 45 min.	1350	80 % of 1 RM	0.51 (0.20)	Stopwatch: 6.1-m course	97 %	87	7
*Liu-Ambrose (2004)* [31]	Progressive high- intensity resistance training initially set at 50–60 % of 1RM (two sets of 10–15 repetitions) progressing to 75-85 % of 1 RM (two sets of 6–8 repetitions)	13 weeks, 2x p/w 50 min.	1300	Progressing from 50-60 % to 75–85 % of 1RM	1.02 (0.25)	Stopwatch: 5-m	85,4 % interv.	79	5
							78.8 % control		
*Lustosa (2011)* [36]	Supervised lower limbs exercises with open chain ankle weights exercises and closed chain body weight exercises	10 weeks, 3x p/w, 60 min.	1800	50-70 % of 1 RM	1.24 (0.14)	Stopwatch: 10-m (accelerated)	-	72	7
*Persch (2009)* [35]	Supervised progressive lower limb strength training	12 weeks, 3x p/w, (estimated at 50 min.)	+/−1800	10-12 maximal reps.	1.23 (0.07)	6-camera motion analysis system (Vicon)	93 %	61	6
*Cress (1999)* [34]	Supervised combined endurance and strength training	6 months, 3x p/w for 60 min.	4860	75-80 % intensity (1 RM and HRR)	1.46 (0.20)	Stopwatch: 20-m course	80.5 %	76	5
*PROGRESSIVE RESISTANCE + BALANCE TRAINING*									
*Freiberger (2012)* [32]	*1)(SB)Strength and balance group:* Progressive upper and lower body strength, balance -, and motor coordination training	16 weeks, 2x p/w; 60 min.	1920	Progressive exertion according BORG scale (not specified)	0.95 (0.22)	Stopwatch: 8-m course (accelerated)	83 % attended ≥ 24 of 32 sessions	76	8
*Yang (2011)*	Personalized home balance and strength exercise program (Based on Otago Exercise Program)	6 months, 5x p/w for +/− 20 min. and daily graduated walking program	2700	Progressive adjustments at 1, 4 and 8 weeks after the baseline	1.02 (0.26)	Stopwatch: 6-m (accelerated)	44.1 % 5x p/w	71	7
							39 % 3-4x p/w		
							13.6 % <2x p/w		
*Arai (2007)* [37]	Supervised progressive resistance training and balance training according to ACSM guidelines	3 months, 2x p/w for 90 min.	2430	65-75 % of 1 RM, 10–15 reps.	1.28 (0.24)	Stopwatch: 10-m (accelerated)	-	74	5
*Gine-Garriga (2010)* [38]	Overload functional circuit training focused at functional balance and lower body strength	12 weeks, 2x p/w; 45 min.	1080	Strength training at perceived exertion of 12–14 on the BORG scale increasing from 6–15 reps. Increasing difficulty in balance exercise	0.82 (0.04)	Stopwatch: 8-m couse (accelerated)	90 % interv.	84	6
							76 % control		
*PROGRESSIVE RESISTANCE + BALANCE + ENDURANCE TRAINING*									
*Baker (2007)* [40]	Supervised exercise: high-intensity progressive resistance training 3 days per week, moderate-intensity aerobic training 2 days per week, and progressive balance training 1 day per week	10 weeks, 3 to 4 h per week divided over 3 days	5400-7200	80 % of 1RM. Aerobic training: rating of perceived exertion 11 to 14 (20) on the BORG scale	1.23 (0.28)	Ultrasonic transmitter/ receiver over 2-m	90 % (excluding dropouts)	77	7
*Barnett (2003)* [39]	Supervised exercise consisting of balance, coordination, endurance and strength training + home exercise program based on class content	4 periods over 1 year 1x p/w; 60 min. (37 lessons)	2220	Complexity, speed and resistance were steadily increased over de 4 periods	0.95 (0.30)	Stopwatch: 6-m course	33.7 % attended ≥ 30 of 37 sessions	75	8
*Freiberger (2012)* [32]	*2)(FG)Fitness Group*; Strength-, balance -and endurance training	16 weeks, 2x p/w; 60 min.	1920	Progressive in duration and exertion according BORG scale (not specified)	0.98 (0.18)	Stopwatch: 8-m course (accelerated)	83 % attended ≥ 24 of 32 sessions	76	8
*Freiberger (2007)* [30]	Fitness intervention: group and home-based strength, flexibility-, balance and motor coordination-, and endurance training	16 weeks, 2x p/w; 60 min.	1920	Not described	1.40 (0.30)	Stopwatch: 8-m (accelerated)	84 %	76	7
*Doi (2013)* [41]	Supervised multicomponent exercise including aerobic exercise, balance-, strength- and gait training	6 months, 2x p/w for 90 min.	4860	Aerobic exercise and gait-training at 60 % max HR.	1.46 (0.20)	Tri-axial accelerometer: 5-m (accelerated)	86.9 %	76	5
*MULTIMODAL TRAINING*									
*Freiberger (2012)* [32]	*3)(MG)Multifaceted group:* Strength and balance, cognition training and fall risk education	16 weeks, 2x p/w; 60 min.	1920	Progressive exertion according BORG scale (not specified)	0.98 (0.20)	Stopwatch: 8-m course (accelerated)	83 % attended ≥ 24 of 32 sessions	76	8
*Kim (2011)* [42]	Weight bearing exercise, chair exercise, resistance band exercise, ball exercise, walking ability training	3 months, 2x p/w 60 min.	1620	Not described	1.10 (0.30)	Stopwatch: ?	77,4 % (≥15 of 24 sessions)	79	7
*Freiberger (2007)* [30]	*Supervised Psychomotor intervention*: strength-, balance-, motor coordination-, competence-, and perceptual training	16 weeks, 2x p/w; 60 min.	1920	Not described	1.40 (0.30)	Stopwatch: 8-m (accelerated)	84 %	76	7
*Lazowski (1999)* [28]	Supervised multicomponent; strength, balance, flexibility, mobility and function	4 months, 3x p/w 45 min.	2430	Self-paced progressive resistance	0.69 (0.28)	Stopwatch: 7-m (accelerated)	85-87 % interv.	80	6
							79 % control		
*Hartmann (2009)* [43]	Aerobic exercises, progressive resistance strength training and stretching exercises + additional foot gymnastic exercises at the end of the training session and a 10-min foot gymnastics home-program daily	12 weeks, 2x p/w supervised 50 min. + 10 min. daily at home	1200+ 840 = 2040	Resistance training 2–3 sets of 12 reps. at an intensity of ‘hard; to ‘very hard’(16–18) on the BORG scale	1.33 (0.19)	DynaPort1MiniMod; tri-axial accelerometer system over 24-m	All subjects completed 24 sessions within 16 weeks	76	6
*DANCE/ RHYTHMIC COMPONENT*									
*Trombetti (2010)* [44]	Supervised progressive multi-task exercises, rhythmic walking	6 months, 1x p/w for 60 min.	1620	Progressing difficulty of exercises	1.04 (0.19)	GAITRite:10-m long electronic gait mat	78 %	76	6
*Granacher (2012)* [45]	Salsa dance training with a dance partner	8 weeks, 2x p/w; 60 min.	960	Increasing music tempo: 50- > 70 BPM	1.34 (0.20)	GAITRite: 10-m long electronic gait mat	92,5 %	71	6
*Liu-Ambrose (2004)* [31]	Agility training; ball games, relay races, dance movements, and obstacle courses	13 weeks, 2x p/w 50 min.	1300	Not described	1.02 (0.19)	Stopwatch: 5-m	85,4 % interv. 78.8 % control	79	5
*STRETCHING*									
*Kerrigan (2003)* [17]	Hip-stretching exercise at home	10 weeks, 2x p/d; 5 min.	700	4 sets of 30 s.	1.19 (0.18)	6-camera motion analysis system (Vicon)	94 %	?	7
*Watt (2011)* [18]	Daily hip flexor stretching program, which was supervised twice weekly by 2a rehabilitation clinician	10 weeks, 2x p/d 4 min. home program, 2x p/w supervised	560	2 sets of 60 s.	1.31 (0.25)	10-camera motion analysis system (Vicon 624)	91 %	73	5
*Watt (2011)* [18] *Frail Elderly*	Daily hip flexor stretching program, which was supervised twice weekly by a rehabilitation clinician	10 weeks, 2x p/d 4 min home program, 2x p/w supervised	560	2 sets of 60 s.	1.15 (0.2)	10-camera motion analysis system (Vicon 624)	91 %	77	5
*BALANCE*									
*Wolf (2006)* [48]	Tai Chi training supplemented with home- based exercise	48 weeks, 2x p/w; 60–90 min.	5760-8640	Progressive duration of 60–90 min	1.01 (0.48)	Stopwatch: 10-m (accelerated)	-	81	7
*Halvarsson (2011)* [47]	Individually adjusted, progressive and specific balance group training	3 months, 3x p/w; 60 min.	2430	Progressing demands on the postural control system (5 levels)	1.11 (0.24)	GAITRite: 8-m long electronic gait mat	87 %	76	7
*REMAINING*									
*Granacher (2013)* [49]	Core stability training at moderate intensity	9 weeks, 2x p/w; 60 min	1080 min	Progressively, individually increased (lever lengths, ROM, movement velocity, level of stability)	1.41 (0.14)	10-m-long electronic gait mat, GAITRite	92 %	71	6
*Tiedeman (2013)* [50]	Iyengar-style yoga	12 weeks, 2x p/w; 60 min + 2x p/w 10–20 min at home	1800	Gradually increasing difficulty (time, balance) of postures	1.54 (0.23)	Stopwatch: 4-m (accelerated) from SPPB	83 %	68	8

Four interventions (two balance interventions, a yoga intervention and a core stability intervention) could not be subjected to a meta-analysis because less than three similar interventions were available. Results from six types of exercise interventions for older adults could be subjected to meta-analyses: progressive resistance training, progressive resistance-, and balance training, progressive resistance-, balance-, and endurance training, multimodal exercise (other than a combination of progressive resistance-, balance-, and endurance training), interventions with a rhythmic component, and specific stretching exercises. Specific stretching techniques with regard to improving gait, are targeting the range of motion in hips and ankles. The hypothesis is that a larger range of motion in these joints improves step length and thereby gait speed [17, 18].

### The meta-analyses

#### The effect of progressive resistance training on preferred gait speed (Fig. 2A)

**Fig. 2:**
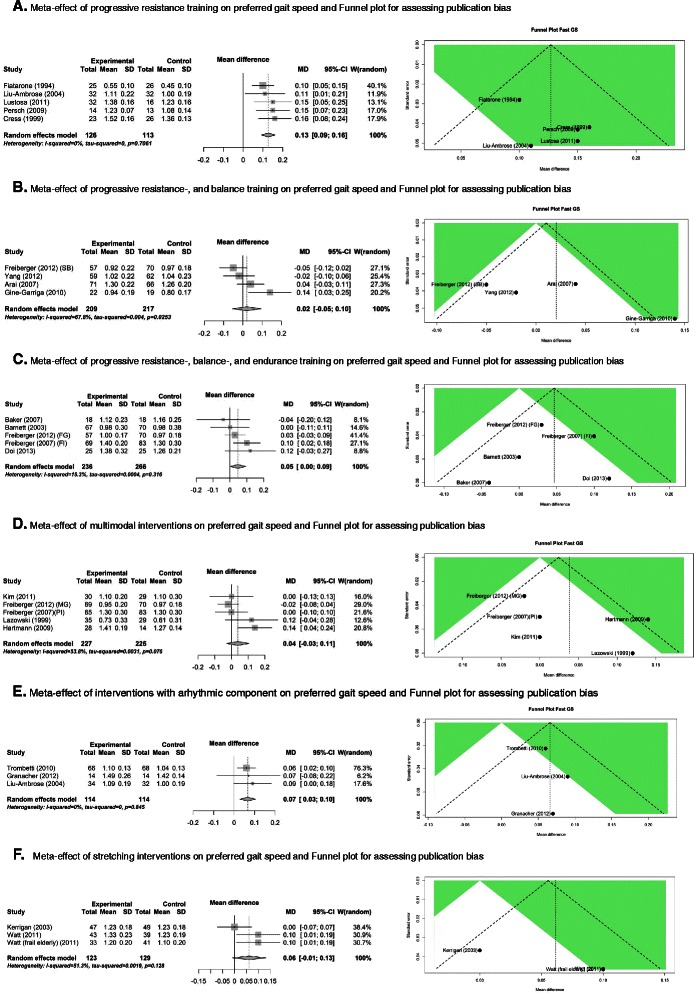
Forest-, and Funnel plots for the six meta-analyses. Meta-effect of progressive resistance training on preferred gait speed and Funnel plot for assessing publication bias. **a**. Meta-effect of progressive resistance-, and balance training on preferred gait speed and Funnel plot for assessing publication bias. **b**. Meta-effect of progressive resistance-, balance-, and endurance training on preferred gait speed and Funnel plot for assessing publication bias. **c**. Meta-effect of multimodal interventions on preferred gait speed and Funnel plot for assessing publication bias. **d**. Meta-effect of multimodal interventions on preferred gait speed and Funnel plot for assessing publication bias. **e**. Meta-effect of interventions with a rhythmic component on preferred gait speed and Funnel plot for assessing publication bias. **f**. Meta-effect of stretching interventions on preferred gait speed and Funnel plot for assessing publication bias

Five trials were included in the first meta-analysis [29, 31, 34–36]. There is a significant positive meta-effect of 0.13 [CI 95 % 0.09-0.16] m/s difference between experimental and control groups. The insignificance of tau-squared indicates acceptation of homogeneity of variances. There are no influential studies present, and the Funnel plot shows no indication of publication bias. The size of the meta-effect indicates a substantial clinically meaningful change (>0.10 m/s). All five interventions in this meta-analysis have a conclusive positive effect on preferred gait speed.

#### The effect of progressive resistance-, and balance training on preferred gait speed (Fig. 2B)

Four trials were included in the second meta-analysis [32, 33, 37, 38]. There is an insignificant positive meta-effect of 0.02 [CI 95 % -0.05-0.10] m/s difference between experimental and control groups . The significance of tau-squared indicates rejection of homogeneity of variances. There are no influential studies present, and the Funnel plot shows no indication for publication bias. The only study with a significant positive effect is the progressive functional circuit training by Giné-Garriga et al. [38].

#### The effect of progressive resistance-, balance-, and endurance training on preferred gait speed (Fig. 2C)

Five trials were included in the third meta-analysis [30, 32, 39–41]. There is a significant positive meta-effect of 0.05 [CI 95 % 0.00-0.09] m/s difference between experimental and control groups. The insignificance of tau-squared indicates acceptation of homogeneity of variances. There are three influential studies present [30, 32, 41], when one of those studies is omitted, the meta-effect becomes insignificant. The Funnel plot shows no indication of publication bias. The size of the meta-effect indicates a small clinically meaningful change (≥0.05 m/s).

#### The effect of multimodal exercise other than a combination of progressive resistance-, balance-, and endurance training on preferred gait speed (Fig. 2D)

Five studies were included in the fourth meta-analysis [28, 30, 32, 42, 43]. There is an insignificant positive meta-effect of 0.04 [CI 95 % -0.03-0.11] m/s difference between experimental and control groups. The insignificance of tau-squared indicates acceptation of homogeneity of variances. There are no influential studies present, and the Funnel plot shows no indication of publication bias.

#### The effect of interventions with a rhythmic component on preferred gait speed (Fig. 2E)

Three studies were included that had a rhythmic component in their intervention [31, 44, 45]. The comparable element within those interventions, is walking or dancing while keeping time to music or rhythm. There is a significant positive meta-effect of 0.07 [CI 95 % 0.03-0.10] m/s difference between experimental and control groups. The insignificance of tau-squared indicates acceptation of homogeneity of variances. There are no influential studies present, and the Funnel plot shows no indication of publication bias. The size of the meta-effect lies in between a small and substantial clinically meaningful change.

#### The effect of stretching on preferred gait speed (Fig. 2F)

Three studies that performed a stretching intervention were included in the last meta-analysis [17, 18, 46]. There is an insignificant positive meta-effect of 0.06 [CI 95 % -0.01-0.13] m/s difference between experimental and control groups. The insignificance of tau-squared indicates acceptation of homogeneity of variances. The study of Kerrigan et al. [17] is influential, when this study is omitted, the meta-effect becomes significant. Furthermore, the Funnel plot shows an indication of publication bias.

An overview of the evidence from the meta-analyses and is shown in Table 3.

**Table 3:** Overview of the evidence from the meta-analyses of different types of exercise interventions

Exercise Intervention	Interventions	Meta-effect	Homogeneous	Influential study	Unbiased
Progressive resistance training	5	YES	YES	NO	YES
Progressive resistance training + Balance	4	NO	NO	NO	YES
Progressive resistance training + Balance + Endurance	5	YES	YES	YES	YES
Multimodal	5	NO	YES	NO	YES
Rythmic	3	YES	YES	NO	YES
Stretching	3	NO	YES	YES	NO
Balance	2	-	-	-	-
Other (yoga/Core stability)	2	-	-	-	-

### Sub-analyses

The mean baseline gait speed in sub-analysis C (progressive resistance training + balance + endurance) was significantly higher in the two interventions with the most improvement in gait speed, other sub-analyses revealed no significant differences.

### Studies not included in the meta-analyses

Four of the studies could not be included in a meta-analysis, because there were less than three similar interventions; two balance-, a core-stability-, and a yoga intervention. A progressive balance group training for community-dwelling older adults did not have significant effect on preferred gait speed (p = 0.12) [47]. The second study with a balance intervention performed an intense Tai Chi training for transitionally frail older adults. The intervention group improved preferred gait speed over four to eight months, as well as the control group, however there was a significant difference between groups in favour of the intervention group (p = 0.02) [48]. At 12 months, this advantage disappeared (p = 0.19). The core stability training for community-dwelling older adults with a mean age of 71 years, showed significantly improved preferred gait speed compared to the control group after nine weeks of core stability training (p = 0.02) [49]. Finally, the Iyengar yoga intervention showed significant positive effects on the 4-m walk time in healthy community-dwelling older adults (mean difference: −0.50 (−0.72 to −0.28); p < .001) [50].

### Long-term follow up analyses

Four studies performed a long term follow-up analysis, after the post intervention measurements (Table 4). Halvarsson et al. (2013) did not find any significant long term effects of progressive group balance training on preferred gait speed [51]. Kim et al. [42] found a significant group by time interaction for preferred walking speed (F = 13.03, p < 0.01), three months after the completion of a multimodal exercise program, with significantly greater increase in the exercise group. Functional circuit training accomplished significant improvements in preferred gait speed (*p* = .002) that were maintained from baseline to the follow up; 6 months after post-intervention measurements in the study of Gine-Garriga et al. [52]. Freiberger et al. [32] performed a mixed-effects regression analysis that revealed significant greater improvements (p < 0.05) in preferred gait speed in the Strength and Balance group (mean difference −0.42 (CI:–0.78 to −0.06)) and the Fitness group (mean difference: −0.50 (CI:–0.87 to −0.13) at 12 months post-intervention.

**Table 4:** Long term effects on preferred gait speed

Study		Baseline		Post-intervention		+3-months follow up		+6-month follow up		+12- months follow up	
		N	m/s (SD)	N	m/s (SD)	N	m/s (SD)	N	m/s (SD)	N	m/s (SD)
Halvarsson (2013) [53]	Intervention	38	1.11 (0.23)	34	1.19 (0.17)	-	-	32	1.16 (0.19)	30	1.15 (0.24)
	Control	21	1.09 (0.22)	21	1.10 (0.23)	-	-	20	1.08 (0.22)	18	1.02 (0.28)
Kim (2011) [42]	Intervention	31	1.10 (0.30)	30	1.10 (0.20)	30	1.20 (0.20)	-	-	-	-
	Control	30	1.20 (0.20)	29	1.10 (0.20)	29	1.10 (0.30)	-	-	-	-
Gine-Garriga 2013	Intervention	26	0.82 (0.19)	22	0.94 (0.19)	-	-	18	0.88 (0.19)	-	-
	Control	25	0.82 (0.17)	19	0.80 (0.17)	-	-	7	0.81 (0.17)	-	-
Freiberger (2012) [32]	Intervention SB	63	0.95 (0.22)	57	0.92 (0.22)	-	-	53	0.93 (0.22)	49	0.98 (0.20)
	FG	64	0.98 (0.18)	57	1.00 (0.17)	-	-	54	0.97 (0.19)	48	1.03 (0.16)
	MG	72	0.98 (0.20)	69	0.95 (0.20)	-	-	69	0.93 (0.20)	57	0.93 (0.19)
	Control	78	0.95 (0.27)	70	0.97 (0.18)	-	-	64	0.93 (0.20)	51	0.95 (0.24)

## Discussion

Preferred gait speed is an important outcome of exercise interventions for older adults, because increased preferred gait speed is associated with increased survival rates in older adults [4]. The meta-analyses have identified two types of exercise interventions that show significant and clinically meaningful meta-effects on preferred gait speed in older adults: progressive resistance training and exercise with a rhythmic component.

When providing resistance training, the focus of improving gait speed is on underlying impairments in muscle strength. For example, a reduction in ankle plantar flexion power limits forward progression of the body, and diminishes momentum of the leg swing, thus reducing step length. This can lead to a redistribution of muscle moment and power in knees and hips [53].

Because there is limited time to effectively exercise with this target population it is important for clinical practice to learn if we should focus on progressive resistance training alone, or also invest time in another type of exercise modality that contributes to the results. According to the results from this study, and in contrast to our hypothesis, the addition of balance training or endurance-, and balance training does not contribute to the significant positive effects of progressive resistance training. The effects of endurance training remain unforthcoming is this study; an improvement in endurance may not be exposed during a short gait speed test. The problem with balance training may be, that it is not sufficiently task-oriented. As a result, no transfer of balance skills to gait performance are present. This assumption is supported by the study of Freiberger et al. [30]; this was the only balance intervention within this study that did have a significant positive effect on preferred gait speed. The balance and motor coordination training included standing balance, dynamic weight transfers, stepping strategies, motor control when performing ADLs, motor control under time pressure and sensory awareness.

The difficulty with the studies that investigate multimodal exercise is, that you cannot isolate the effect of the individual components. The multimodal programs may have too many components to produce individual effects of the components. The only intervention with significant positive results in the multi-modal arm of the meta-analyses is the study of Hartman et al. [43]. They combined aerobic exercises, progressive resistance training, ankle stretching exercises and foot gymnastics targeting the earlier mentioned ankle plantar flexion weakness. Lower extremity stretching exercises alone does not seem to have impact on gait speed, as shown in the stretching arm of the meta-analyses.

A promising type of intervention for improving preferred gait speed are interventions with a dance- or rhythmic component, like salsa dancing and rhythmic walking. The corresponding element within those interventions, is walking or dancing while keeping time to music or rhythm. This was a small meta-analysis consisting of three studies. However, it is an interesting finding, that gives rise to future research on this type of interventions. In recent years, gait is considered a higher cognitive function rather than a simple automatic motor activity [54]. Safe walking and adapting gait to the environmental conditions, requires the processing and rapidly updating of visual, vestibular and proprioceptive information. Possibly, keeping time to music or rhythm, is a task performance that trains higher cognitive functions.

The progressive resistance training seems to influence preferred gait speed in frail older adults, with a mean baseline gait speed of 0.51 m/s, as well as in more fit older adults with a mean baseline gait speed of 1.46 m/s. The connection between the improvement in fundamental motor skills and gait speed are not obvious from this study. It could be argued that small gains in strength or endurance could result in larger gait speed improvement in frail older adults, than in healthy older adults. The decline in physical capacity in frail older adults is probably closer to the disability threshold, where small declines can cause major negative impact on daily functioning, and small increases can cause large positive effects on functioning. Further research is needed to clarify the effect of these interventions on health status and daily functioning of frail and healthy elderly, especially on the long term.

The results support the positive findings of Liu and Latham (2009, 2011) investigating the effect of progressive resistance training on physical disability, including evaluations of physical performance. However, they included younger (50 years and older), and more diverse research populations, and sometimes complementary interventions like vitamin supplementation versus placebo tablets [55, 56, 54]. The meta-analysis of Lopopolo et al. [12] found a very small positive change in preferred gait speed of 0.02 m/s resulting from strength training that was not clinically meaningful.

This is the first meta-analysis on the effect of exercise on preferred gait speed in older adults, that only included RCT’s with high level of evidence. Furthermore, because there are so many types of exercise interventions, the differentiation in exercise modalities provides more insight in the effectiveness of specific types or combinations of exercise, to improve preferred gait speed in older adults. Although careful considerations were made, the choice in different sub-analyses, and assignment of interventions may be disputable in a few cases*:* In the first arm of the meta-analyses the study of Cress et al. [34] not only provides progressive resistance training, but aerobic training as well. We included the study is this arm, because it was the only study that combined these two modalities and could not be analysed separately, and moreover, because the equipment that was used for the aerobic training was a stair stepper, and a kayak machine that both also improve leg strength [33]. The study of Freiberger et al. [30] and Doi et al. [41] were included in the third arm of a combination of resistance-, balance-, and endurance training, although the first study also included a flexibility-, and the second also included a gait training component. The main components were however resistance-, balance-, and endurance training, unlike the combinations of interventions in the studies included in the multimodal arm of the analyses.

After applying our search string, there were also articles retrieved with study populations that included older adults of 60 years and older [29, 38, 43]. We decided to include these three articles, because the mean age, and/or baseline gait speed within this articles were no outliers in relation to the data from other included studies. Furthermore, although research populations with specific pathologies were excluded, there is still heterogeneity within research populations of older adults. However, only for the sub-analysis C (progressive resistance-, balance- and endurance training) a significant difference was found for baseline gait speed. The study populations that performed better at post-interventions had higher baseline gait speeds. This could indicate that this type of interventions have more effect on healthier, fitter older adults.

Overall, more studies are needed with comparable interventions to enlarge the body of knowledge on effective exercise modalities, or combinations improving preferred gait speed, and preserving preferred gait speed after the interventions. Furthermore, the influence of variables like group size, instructor, instructions and exercise environment could be important to assess within RCT’s that investigate the effect of specific exercise interventions in older populations. Another important behavioural aspect is how to involve older adults in (preventive) exercise training, how to keep them motivated during the training, and how to inspire them to keep active after the exercise program.

Follow up data is lacking for examining the long term effects of exercise interventions on preferred gait speed. Only four studies collected data as follow up to the post-intervention measurements. Those results are promising; gain in preferred gait speed was maintained three [40], six [36], and 12 months [30] after the interventions were completed. However, only 2 out of 4 studies performed intention to treat analysis [30, 40].

## Conclusions

The preliminary conclusions of these meta-analyses are that progressive resistance training with high intensities seems the most effective exercise modality for improving preferred gait speed. The addition of balance training or balance- and endurance training does not seem to contribute to the positive effect of resistance training. Another promising component that needs further research is exercise with a rhythmic component, possibly training higher cognitive functions that are important for gait. More long-term data is needed to gain knowledge on the course of gait speed over time after interventions have ended, and what is needed to maintain the benefits from training

## Additional files

Additional file 1:
**PRISMA 2009 Checklist.**

Additional file 2:
**Search String Pubmed.**

Additional file 3:
**PEDro scores for the qualitative analysis of the trials.**
