# Supplementary material for: What type, or combination of exercise can improve preferred gait speed in older adults? A meta-analysis
Source: BMC Geriatr. 2015 Jul 1;15:72. doi: 10.1186/s12877-015-0061-9 (PMC4488060; doi:10.1186/s12877-015-0061-9)
Supplement: Additional file 2: — Search String Pubmed. [file 12877_2015_61_MOESM2_ESM.pdf]

Appendix A

Search String Pubmed

The date last searched was 9-12-2013.

Search ((((((((((((((((((((((((((((((((Frail elderly*) OR Aged[MeSH Terms]) OR (Aged, 80 and over[MeSH Terms])) OR frailty)) AND Randomized Controlled Trial[Publication Type]) AND ((((((((Exercise*[MeSH Terms]) OR exercise/physiology*[MeSH Terms]) OR Exercise Therapy*[MeSH Terms]) OR Physical therapy (specialty)[MeSH Terms]) OR Physical therapy modalities[MeSH Terms]) OR Exercise Movement Techniques[MeSH Terms]) OR (Physical Education and Training/methods[MeSH Terms])) OR Activities of Daily Living[MeSH Terms])) AND (((((((((Gait/physiology) OR walking/physiology*) OR mobility) OR gait*) OR gait speed*) OR gait velocity*) OR walking speed*) OR rapid gait*) OR walking*)) AND Humans[Mesh] AND English[lang] AND aged[MeSH])) NOT Peripheral Arterial Disease[MeSH Major Topic]) NOT Stroke[MeSH Major Topic]) NOT Parkinson Disease[MeSH Major Topic]) NOT Bone fractures[MeSH Major Topic]) NOT Osteoarthritis[MeSH Major Topic]) NOT drug effects[MeSH Subheading]) NOT alzheimer disease[MeSH Major Topic]) NOT Multiple sclerosis[MeSH Major Topic]) NOT Chronic obstructive pulmonary disease[MeSH Major Topic]) NOT Arthroplasty, Replacement, Hip[MeSH Major Topic]) NOT Arthroplasty, Replacement, Knee[MeSH Major Topic]) NOT Neoplasms[MeSH Major Topic]) NOT spinal cord injuries[MeSH Major Topic]) NOT cardiovascular diseases[MeSH Major Topic])

LIMITS: Humans, Aged: 65+ years
