# Supplementary material for: What type, or combination of exercise can improve preferred gait speed in older adults? A meta-analysis
Source: BMC Geriatr. 2015 Jul 1;15:72. doi: 10.1186/s12877-015-0061-9 (PMC4488060; doi:10.1186/s12877-015-0061-9)
Supplement: Additional file 3: — PEDro scores for the qualitative analysis of the trials. [file 12877_2015_61_MOESM3_ESM.pdf]

Appendix B: PEDro scores for the qualitative analysis of the trials

| | ***PEDro-scale*** <br> ***Article*** | ***2*** | ***3*** | ***4*** | ***5*** | ***6*** | ***7*** | ***8*** | ***9*** | ***10*** | ***11*** | ***Total*** |
|---|---|---|---|---|---|---|---|---|---|---|---|---|
| 1 | Arai 2007 | y | n | y | n | n | y | n | n | y | y | 5 |
| 2 | Baker 2007 | y | y | y | n | n | y | n | y | y | y | 7 |
| 3 | Barnett 2003 | y | y | y | n | n | y | y | y | y | y | 8 |
| 4 | Beling 2009 (excluded) | y | n | y | n | n | n | n | n | y | y | 4 |
| 5 | Cress 1999 | y | n | y | n | n | n | y | n | y | y | 5 |
| 6 | Doi 2013 | y | n | y | n | n | n | y | n | y | y | 5 |
| 7 | Fiatarone 1994 | y | n | y | n | n | n | y | y | y | y | 6 |
| 8 | Freiberger 2007 | y | n | y | n | n | y | y | y | y | y | 7 |
| 9 | Freiberger 2012 | y | y | y | n | n | y | y | y | y | y | 8 |
| 10 | Gine-Garriga 2010 | y | n | y | n | n | n | y | n | y | y | 5 |
| 11 | Granacher 2012 | y | y | y | n | n | n | y | y | y | y | 7 |
| 12 | Granacher 2013 | y | y | y | n | n | n | y | y | y | y | 7 |
| 13 | Halvarsson 2011 | y | y | y | n | n | y | y | n | y | y | 7 |
| 14 | Hartmann 2005 | y | n | y | n | n | n | y | y | y | y | 6 |
| 15 | Kerrigan 2003 | y | n | y | y | n | y | y | n | y | y | 7 |
| 16 | Kim 2011 | y | y | y | n | n | y | n | y | y | y | 7 |
| 17 | Lazowski 1999 | y | n | n | y | y | y | n | n | y | y | 6 |
| 18 | Liu-Ambrose 2004 | y | n | y | n | n | n | y | n | y | y | 5 |
| 19 | Lord 1996 (excluded) | y | y | n | n | n | n | n | n | y | y | 4 |
| 20 | Lustosa 2011 | y | n | y | n | n | y | y | y | y | y | 7 |
| 21 | Persch 2009 | y | n | y | n | n | n | y | y | y | y | 6 |
| 22 | Tiedeman | y | y | y | n | n | y | y | y | y | y | 8 |
| 23 | Topp 1996 (excluded) | y | n | y | n | n | n | n | n | y | y | 4 |
| 24 | Trombetti 2012 | y | y | y | n | n | y | n | y | y | y | 7 |
| 25 | Watt 2011 | y | n | y | y | n | y | n | n | n | y | 5 |
| 26 | Watt 2011 (Frail) | y | n | y | y | n | y | n | n | n | y | 5 |
| 27 | Wolf 2006 | y | y | y | n | n | y | y | y | y | y | 8 |
| 28 | Yang 2012 | y | y | y | n | n | y | n | y | y | y | 7 |

PEDro items:

2. subjects were randomly allocated to groups (in a crossover study, subjects were randomly allocated an order in which treatments were received)
3. allocation was concealed
4. the groups were similar at baseline regarding the most important prognostic indicators
5. there was blinding of all subjects
6. there was blinding of all therapists who administered the therapy
7. there was blinding of all assessors who measured at least one key outcome
8. measures of at least one key outcome were obtained from more than 85% of the subjects initially allocated to groups
9. all subjects for whom outcome measures were available received the treatment or control condition as allocated or, where this was not the case, data for at least one key outcome was analyzed by “intention to treat”
10. the results of between-group statistical comparisons are reported for at least one key outcome
11. the study provides both point measures and measures of variability for at least one key outcome
